# Efficacy and mechanism of Jiedu Tongluo Tiaogan Formula in treating type 2 diabetes mellitus combined with non-alcoholic fatty liver disease: Study protocol for a parallel-armed, randomized controlled trial

**DOI:** 10.3389/fphar.2022.924021

**Published:** 2022-08-12

**Authors:** Jinghan Xu, Chunli Piao, Yue Qu, Tianjiao Liu, Yuting Peng, Qi Li, Xiaohua Zhao, Pei Li, Xuemin Wu, Yawen Fan, Binqin Chen, Jie Yang

**Affiliations:** ^1^ Guangzhou University of Chinese Medicine, Guangzhou, China; ^2^ Shenzhen Hospital, Guangzhou University of Chinese Medicine (Futian), Shenzhen, China; ^3^ Changchun University of Chinese Medicine, Jilin, China

**Keywords:** jiedu tongluo tiaogan formula, type 2 diabetes, non-alcoholic fatty liver disease, randomized controlled trial, metabolomics, traditional Chinese medicine

## Abstract

**Background:** The incidence of Type 2 diabetes mellitus (T2DM) combined with non-alcoholic fatty liver disease (NAFLD) has risen over the years. This comorbid condition significantly increases the probability of cirrhosis, liver cancer, and mortality compared to the disease alone. The multi-targeted, holistic treatment efficacy of traditional Chinese medicine (TCM) plays a vital role in the treatment of T2DM and NAFLD. Jiedu Tongluo Tiaogan Formula (JTTF), based on TCM theory, is widely used in clinical treatment, and its effectiveness in lowering glucose, regulating lipids, improving insulin resistance, and its pathways of action have been demonstrated in previous studies. However, the mechanism of this formula has not been investigated from a metabolomics perspective. Moreover, high-quality clinical studies on T2DM combined with NAFLD are lacking. Therefore, we aim to conduct a clinical trial to investigate the clinical efficacy, safety, and possible pathways of JTTF in the treatment of T2DM combined with NAFLD using metabolomics techniques.

**Methods:** A total of 98 participants will be recruited to this clinical trial and randomly assigned to either a treatment group (JTTF + conventional basic treatment) or control group (conventional basic treatment) in a 1:1 ratio. Both groups will have received the same lifestyle interventions in the preceding 12 weeks. The primary outcome will be change in visceral fat area and total score on the TCM syndromes efficacy score scale. The secondary outcome will include changes in ultrasound steatosis grade, fibrosis 4 score (FIB-4), metabolic parameters, anthropometric parameters, visceral fat area. In addition, serum and urine samples collected at baseline and at the end of 12 weeks of treatment will be sequentially tested for untargeted and targeted metabolomics.

**Discussion:** This study will evaluate the efficacy and safety of JTTF, as well as investigate the differential metabolites and possible mechanisms of JTTF treatment in T2DM combined with NAFLD. We hypothesize that patients will benefit from JTTF, which may provide strong evidence for the clinical use of JTTF in the treatment of T2DM and NAFLD, leading to the possibility of further mechanistic exploration.

**Clinical Trial Registration:** This clinical trial has been registered in China Clinical Trial Registry (ChiCTR 2100051174).

## 1 Introduction

The global burden of diabetes continues to rise annually among the general population with a predicted increase from 10.5% (536.6 million people) in 2021 to 12.2% (783.2 million) by the year 2045 in people aged 20–79 years (Sun et al., 2022). According to the epidemiological survey conducted by Li et al. (Li et al., 2020), diabetes is prevalent in 11.2% of the Chinese population. Diabetes and related complications significantly affect quality of life, mortality and morbidity, and contributes to greater economic burden on individuals and society at large (American Diabetes Association, 2018). Non-alcoholic fatty liver disease (NAFLD) is one of the most common chronic liver diseases worldwide and includes various pathological states such as simple steatosis and non-alcoholic steatohepatitis (NASH). The disease is initially accompanied by fat accumulation and may gradually progress to other diseases such as liver fibrosis, cirrhosis, or hepatocellular carcinomas (Chalasani et al., 2018). In the absence of any intervention for NAFLD, human health is endangered and the risk of death increases by at least 71% (Tilg and Targher, 2021). Studies have shown that the prevalence of NAFLD in patients with T2DM is greater than 55% (Younossi et al., 2019), which is 5–9 times higher than the incidence in the general population. Conversely, individuals with NAFLD have a significantly higher risk of developing diabetes than those without NAFLD, which is more than twice as high as that of healthy individuals (Mantovani et al., 2018). More than 60% of patients were found to have abnormal glucose metabolism, leading to a 47.3% probability of diabetes (Buzzetti et al., 2016); thus NAFLD may be an early predictor of T2DM (Lee et al., 2019).

The pathogenesis of T2DM and NAFLD is complex and not yet fully understood. However, it is widely accepted that the two diseases share a common pathogenesis, insulin resistance (IR), both as a cause and a consequence of T2DM combined with NAFLD (Park et al., 2013). Clinical trials developing drugs to treat NAFLD have also focused on the discovery of insulin sensitizers (Ibrahim et al., 2013). Many of the same changes have been shown to occur in the development of both diseases, including abnormal glucose and lipid metabolism, genetic susceptibility, environmental influences, and lifestyle changes (Saponaro et al., 2015). NAFLD is usually treated as a benign disease and neglected for management and treatment. However, when it occurs in combination with diabetes, it exacerbates imbalances in glucolipid metabolism, and abnormalities in glucose and lipid metabolism interact to affect each other. Moreover, diabetes increases the risk of cirrhosis and mortality in patients with NAFLD, while NAFLD increases the incidence of cardiovascular disease and all-cause mortality in patients with T2DM (Radaelli et al., 2018). In the presence of abnormalities in the relevant metabolic and anthropometric indicators, clinical screening for disease should be performed promptly to exclude them. The occurrence of this comorbidity is usually accompanied by abnormalities in glucolipid metabolism-related indicators such as glucose, lipids, and insulin levels (Ziolkowska et al., 2021). Also, some anthropometric indicators are closely associated with the occurrence and development of comorbidity, represented by WHtR (Swainson et al., 2017). Therefore, it makes sense to adopt a holistic approach to the treatment of these metabolic diseases with highly related pathogenesis and pathological manifestations.

Changes in molecular products in the human body can precede the appearance of clinical symptoms (Wu et al., 2019); hence, metabolomics has been gradually applied in recent years to explore disease diagnosis, pathogenesis, and risk assessment (Roberts et al., 2014; Newgard, 2017, 2017; Wishart, 2019; Schmidt et al., 2021). This technique may also identify biomarkers for the early diagnosis of a disease (Esperanza et al., 2020; Cui et al., 2021) or explore metabolic differentials in the action of Chinese and Western drugs (Tao et al., 2019; Wu et al., 2019; Xiao et al., 2020; Xu et al., 2020; Luo et al., 2021; You et al., 2021). Further, it has applications for assessing the variability of molecular metabolic products in patients with different traditional Chinese medicine (TCM) syndromes of the same disease (Guo et al., 2021; Shan et al., 2021). Other studies have applied metabolomic techniques to the diagnosis and mechanistic exploration of diabetes and its complications (Jimenez-Luna et al., 2020; Wang S. et al., 2021; Zhang F. et al., 2021; Tan et al., 2021), and to NAFLD (Masoodi et al., 2021; Piras et al., 2021). Therefore, it is feasible and relevant to study the treatment of this comorbid disease at a molecular level.

The current Western medical treatment of T2DM combined with NAFLD is mainly aimed at reducing fat accumulation in the liver and delaying the onset of inflammation and fibrosis (Ks et al., 2019), and relies on lifestyle management, the application of glucose- and lipid-lowering drugs, and the implementation of bariatric surgery. Glucose management is particularly important for inhibiting the development of liver fibrosis (Nakahara et al., 2014). Lifestyle modification is a basic form of treatment for patients with T2DM and NAFLD. Reducing body mass index (BMI) can effectively improve liver function and insulin resistance levels (Al-Jiffri et al., 2013). However, non-pharmacological therapies often show low compliance in the patient population and are difficult to adhere to. Although there are currently no Food and Drug Administration (FDA)-approved drugs for NAFLD or NAFLD combined with type 2 diabetes (Ferguson and Finck, 2021), many emerging drug therapies such as targeted therapies are in development (Negi et al., 2022). The continuous clinical exploration of drugs for the treatment of NAFLD in combination with T2DM shows the high incidence of this comorbidity and the necessity of treatment. There are many glucose-lowering drugs also used in the treatment of NAFLD in the clinic, such as glucagon-like peptide-1 (GLP-1), Thiazolidinediones, Metformin, which have good effect on fat reduction and glucose reduction (Negi et al., 2022). However, there are still some limitations, for example, metformin has a weak effect on hepatic fat; thiazolidinediones may show weight gain and female fractures despite their insulin-sensitive effects; GLP-1 has a significant weight loss effect but is often accompanied by gastrointestinal side effects. In summary, additional therapeutic strategies are urgently required to treat this complicated disease.

TCM treats diseases from a holistic viewpoint, harmonizing the organs of the whole body in a comprehensive manner. This medicine system has multi-component, multi-target, multi-pathway, prospective, and stable characteristics, and is especially effective in treating complex and variable chronic diseases such as T2DM (Wang X. et al., 2021). Chinese medicine can improve IR and glucolipid metabolic function, among other effects, by regulating the inflammatory response and oxidative stress capacity, and improving the structure of the intestinal flora, thereby treating T2DM combined with NAFLD. It not only improves the clinical index of patients but also alleviates clinical symptoms for the purpose of prevention and treatment. For example, the Chinese botanical drugs such as Shengmaiyin and Ganmai Dazao Tang (Fan et al., 2018; Li et al., 2019), and other appropriate Chinese medicine techniques such as acupuncture and acupuncture point burial are widely used in clinical practice to assist in lowering sugar and reducing fat (Li et al., 2021; Wang et al., 2022).

Jiedu Tongluo Tiaogan Formula (JTTF) is a clinically effective formula based on TCM theory, consisting of the dried rhizome of *Coptis chinensis* Franch [Ranunculaceae; Coptidis rhizome], the dried rhizome of *Rheum palmatum* L [Polygonaceae; Rhei radix et rhizoma], the dried root of *Astragalus mongholicus* Bunge [Fabaceae; Astmgali radix], the dried rhizome of *Salvia miltiorrhiza* Bunge [Lamiaceae; Salviae miltiorrhizae radix et rhizoma], the dried root of *Bupleurum chinense* DC [Apiaceae; Radix bupleuri], at the weight ratio of 15:9:15:15:10. This formula is involved in detoxifying and regulating the draining function of the liver to eliminate the “toxic evil” in the body for disease prophylaxis and treatment. The effectiveness of this formula in lowering blood glucose, regulating blood lipids, promoting insulin secretion, and improving IR has been verified in many basic and clinical trials.

The main components of JTTF have been analyzed by High Performance Liquid Chromatography (HPLC) in our previous study (Zhang Q. et al., 2021), which can be seen in Supplementary Material S1. On the basis of reference standard data, chromatographic elution behavior, chemical composition data, mass fragmentation pattern and HPLC results, the chemical components of JTTF were identified as chlorogenic acid, anthocyanin-7-glucoside, tannic acid B, aloe rhodopsin and haragoside. By means of the validation of network pharmacology and in vitro experiments, it proves that JTTF plays an important role in promoting insulin secretion and regulating glucolipid metabolism by acting on the PI3K-Akt pathway, an important pathway for insulin signaling and glucose regulation (Zhang Q. et al., 2021). Related research has demonstrated that JTTF can inhibit the expression of inflammatory factors associated with endoplasmic reticulum stress, thereby alleviating this condition (Jiang et al., 2018), improving IR, and increasing insulin sensitivity (Liu et al., 2021). At the same time, it can activate the IRE1α/JNK pathway, which is closely related to IR and pancreatic β-cell apoptosis to reduce the apoptosis of pancreatic islet cells and prevent and treat T2DM (Piao et al., 2018). Studies showed that blood glucose and lipid levels, as well as IR levels of diabetic ZDF rats were significantly improved by high-dose JTTF (Liu et al., 2021). Related clinical studies have shown that JTTF may reduce glycated hemoglobin, blood glucose, and inflammatory factor levels, regulate lipid and insulin resistance levels in T2DM patients (Jin et al., 2020), and have significant efficacy in reducing liver function, BMI, waist circumference, and TCM symptom scores in T2DM combined with NAFLD patients (Gong, 2017).

Herein we investigate the clinical efficacy and safety of JTTF for the treatment of T2DM combined with NAFLD. It is necessary to design and conduct this trial study as the mechanism of action of this formula has not been explored at the molecular level.

## 2 Methods and analysis

### 2.1 Study objectives

#### 2.1.1 Primary objective

The primary objective of this clinical trial is to assess the efficacy and safety of JTTF for the treatment of T2DM combined with NAFLD. We hypothesized that JTTF combined with conventional Western medicine would be more effective than the single use of conventional Western medicine alone in reducing fat accumulation, inhibiting liver fibrosis, regulating blood lipids, lowering blood glucose levels, and alleviating the clinical symptoms of patients with T2DM and NAFLD.

#### 2.1.2 Secondary objective

The secondary objective of this study was to identify differential metabolites in the serum and urine of patients before and after JTTF treatment to determine possible relevant metabolic pathways and routes of action in T2DM combined with NAFLD. Furthermore, we explored the regulatory effects and mechanisms of JTTF action on various aspects including lipid and glucose regulation at the metabolic level.

### 2.2 Study design

This study is designed as a 12-weeks, single-center, double-armed, parallel, randomized, controlled clinical trial. A total of 96 eligible participants will be recruited from Shenzhen Hospital of Guangzhou University of Chinese Medicine (Futian) and randomized in a 1:1 ratio into two groups to receive basic treatment. As a supplement to the clinical study, participants’ fasting serum and urine samples will be subjected to untargeted and targeted metabolomic assays. Liquid chromatography–mass spectrometry (LC-MS) will be used to compare metabolites before and after treatment in the experimental and control groups. Basic analysis of metabolites, multivariate statistical analysis, pathway enrichment analysis, and topological analysis of the screened differential metabolites will be performed.

**FIGURE 1:**
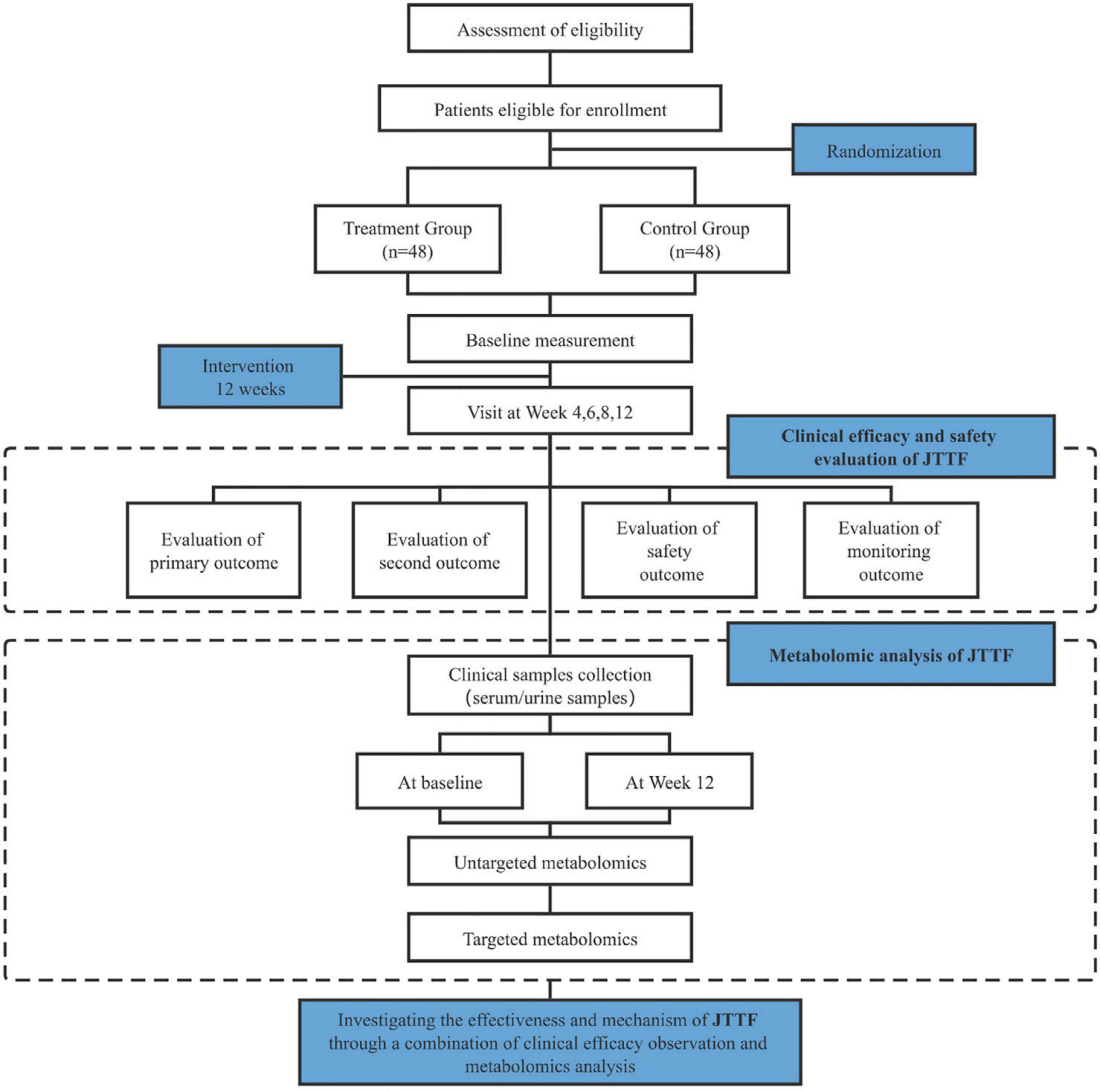
Flow chart of the clinical trial.

All patients should have voluntarily participated and signed an informed consent form with good compliance. Informed consent will be obtained from all participants prior to enrollment. A flowchart and schedule of the clinical trial is shown in Figure 1 and Table 1, respectively. The protocol was designed according to the Standard Protocol Items: Recommendations for Interventional Trials (SPIRIT) guidelines (Chan et al., 2013) and the Declaration of Helsinki. The SPIRIT checklist is shown in Supplementary Material S2.

**TABLE 1:** Schedule of the clinical trial.

Project time	Screening/baseline		Treatment			
	-14–0 days	0±2 days	Visit 1 4weeks± 2days	Visit 2 6weeks± 4days	Visit 3 8weeks± 6days	Visit 4 12weeks± 7days
Eligibility screening						
Inclusion/exclusion criteria	**✓**					
Informed consent form	**✓**					
Allocation	**✓**					
General information	**✓**					
Family history	**✓**					
Medical history	**✓**					
Drinking history	**✓**					
Physical examination^a^	**✓**	**✓**	**✓**	**✓**	**✓**	**✓**
Vital signs^b^	**✓**	**✓**	**✓**	**✓**	**✓**	**✓**
Ultrasound of liver	**✓**					**✓**
bioelectrical impedance analysis						
visceral fat area		**✓**				**✓**
subcutaneous fat area		**✓**				**✓**
Electrocardiography	**✓**			**✓**		**✓**
Laboratory examination						
Routine blood test		**✓**		**✓**		**✓**
Routine urine test		**✓**		**✓**		**✓**
Fasting blood sugar		**✓**		**✓**		**✓**
2-h plasma glucose		**✓**				**✓**
Glycated hemoglobin		**✓**				**✓**
Fasting insulin		**✓**				**✓**
Blood lipids		**✓**		**✓**		**✓**
Liver function		**✓**		**✓**		**✓**
Renal function		**✓**		**✓**		**✓**
Traditional Chinese medicine symptoms		**✓**	**✓**	**✓**	**✓**	**✓**
Fibrosis 4 Score		**✓**				**✓**
Homeostasis model assessment-insulin resistance		**✓**				**✓**
homeostatic model assessment for β-cell		**✓**				**✓**
Metabolomics test^c^						
Serum sample		**✓**				**✓**
Urine sample		**✓**				**✓**
Adverse event			**✓**	**✓**	**✓**	**✓**
Prescription		**✓**	**✓**	**✓**	**✓**	**✓**
Combined drugs		**✓**	**✓**	**✓**	**✓**	**✓**
Summary of the analysis						**✓**

### 2.3 Ethics and dissemination

This clinical trial has been approved by the Ethics Committee of Shenzhen Hospital of Guangzhou University of Chinese Medicine (Futian) (Project number: GZYLL(KY)-2021–025).

Prior to signing the informed consent form, the investigator will provide the patient with a detailed description of the process, potential risks and benefits of participating in the study. Participants will sign an informed consent form and allow the study results to be published in a peer-reviewed journal. All participants’ personal data will be kept confidential during and after the trial. Only authorized researchers have access to all data on participants.

### 2.4 Inclusion criteria

1) All participants must meet the individual diagnostic criteria for T2DM and NAFD. The diagnostic criteria for T2DM (American Diabetes Association Professional Practice Committee, 2022) includes:
  • Fasting plasma glucose (FPG) ≥126 mg/dl (7.0 mmol/L) or 2-h plasma glucose (2-h PG) ≥200 mg/dl (11.1 mmol/L) during an oral glucose tolerance test or HbA1c ≥ 6.5% (48 mmol/mol).
  • Typical symptoms of hyperglycemia (excessive thirst, polyuria, weight loss, hunger, pruritus, or coma) or hyperglycemic crisis plus a random plasma glucose (PG) ≥200 mg/dl (11.1 mmol/L). Patients without typical symptoms of diabetes need to be re-examined for confirmation. Fasting state: no intake of calories for at least 8 h; random plasma glucose: blood glucose at any time of day, regardless of the last meal time (cannot be used to diagnose abnormal fasting blood glucose or abnormal glucose tolerance). The diagnostic criteria for NAFLD (European Association for the Study of the Liver (EASL) et al., 2016) (Chalasani et al., 2018) requires:
  • Evidence of excess liver fat accumulation (hepatic steatosis) on ultrasound imaging.
  • Exclusion of excessive daily alcohol consumption ≥30 g for men and >20 g for women (Ratziu et al., 2010).
  • Acute or chronic viral hepatitis (e.g., hepatitis C), autoimmune hepatitis, hemochromatosis, Wilson’s disease, alcoholic liver disease, drug-induced liver disease, liver cirrhosis, inborn errors of metabolism (e.g., cholesterol ester storage disease), or other causes of chronic liver disease.
2) All participants must meet the diagnostic criteria for heat stagnation syndrome in the liver and stomach in accordance with the Guidelines for Clinical Research of Chinese Medicine (Zheng, 2002) outlined below.
  • Any two main symptoms including dry mouth, bitter and sticky mouth, heaviness in the head and body, and/or obesity
  • Any two secondary symptoms including fullness and distention of the chest or abdomen, upset or irritable demeanor, rapid digestion of food and polyorexia, deep-colored or turbid urine, and/or unpleasant or constipated stools.
  • Tongue condition: red tongue, yellow fur.
• Pulse condition: stringy and rapid pulse or slippery and rapid pulse.
3) Participants should be between the ages of 20 and 75 years.
4) The experimental group should not have taken traditional Chinese medicine for diabetes and NAFD orally for at least 2 weeks before enrollment.

### 2.5 Exclusion criteria

1) Patients with liver or kidney dysfunction: aspartate transaminase [AST] or alanine transaminase [ALT] >2.5 times the normal upper limit or creatinine >115 μmol/L.
2) Patients with chronic diseases, such as hypertension, cardiovascular, cerebrovascular, autoimmune diseases, chronic kidney diseases and psychiatric diseases who cannot follow research procedures.
3) Patients who are using insulin.
4) Allergic constitution or a history of drug allergies.
5) Pregnant and lactating women.
6) Patients who participated in other clinical studies within the preceding three months.

### 2.6 Drop-out criteria

1) Participants who would develop severe, acute conditions (e.g., diabetic ketoacidosis) or special physiological changes (e.g., state of pregnancy) that are not appropriate for this trial.
2) Participants who would show low compliance, such as failing to take their medication regularly or at a rate of less than 80% during the trial.
3) Participants who would have taken drugs prohibited by the study protocol.
4) Participants who would voluntarily withdraw from the trial at any time according to the informed consent form.

### 2.7 Intervention

The control group will receive conventional basic treatment, including health education and oral medications to stabilize blood sugar, blood pressure, and lipid levels. Meanwhile, the experimental group will be given JTTF on the top of the control group. In this study, Chinese herbal medicine concentrated granules (CCMG) will be used to replicate the traditional method of preparing herbal tonics by using herbs as raw materials and modern extraction and concentration techniques. All herbal pellets will be manufactured and supplied by China Resources Sanjiu Pharmaceutical Co. and also tested by their quality management department for compliance with the grade standards of the Chinese Pharmacopoeia (2020 edition). Voucher specimens of all drugs are deposited at the Bureau of Traditional Chinese Medicine, Shenzhen Hospital, Guangzhou University of Chinese Medicine (Futian) (Shenzhen, China). The decoction and packing of botanical drugs are uniformly operated by Kangmei Pharmaceutical Co., Ltd. Smart Pharmacy. CCMG is based on the “Research Guidelines for Single Flavor Chinese Medicine Concentrated Granules” and the “Pharmacopoeia of the People’s Republic of China” (2010 edition). Extracted in a fully enclosed pipeline production line in GMP workshop. All herbs are mixed in the proportions described above, steeped in 10 volumes of pure water for 0.5 h, heated to boiling, decocted for 1 h. The water extract is filtered and the residue is then decocted with 8 volumes of pure water for 40 min. The two liquid extracts are combined, settled and filtered, concentrated to a thick paste, and processed into concentrated granules by spray drying. The process parameters are screened by orthogonal design method according to the composition characteristics of different species. A certain amount of soluble dextrin is added as an auxiliary material to assist the molding. The dry extract ratios of the five raw herbs were 1:6 for *Coptis chinensis*, 1:2.4 for *Rheum palmatum*, 1:10 for *Astragalus mongholicus*, 1:6.7 for *Salvia miltiorrhiza* and 1:6 for *Bupleurum chinense*. The final JTTF is made by combining the individual herbal pellets in the leaching ratio, which is equivalent to 64 g of raw herbs. It is packaged with aluminum-plastic-aluminum composite film (BOPP/AL/CPE) packaging material sealed on four sides. Dissolve the concentrated granules in 200 ml of boiling water and take warm. Take twice daily, half an hour after breakfast and dinner. The observation period will be administered for three months.

Both groups will receive the same health education information on diabetes and NAFLD, assistance, lifestyle interventions, and management. It includes developing a diet of low sugar, low fat, and less refined carbohydrates, controlling the intake of total calories in the diet, helping participants to choose appropriate exercise methods, and encouraging patients to remain relaxed and actively cooperate with treatment. Face-to-face nutritional education will be provided at each follow-up visit.

### 2.8 Outcome assessment

#### 2.8.1 Demographic information

All demographic information will be collected at enrollment, including sex, age, course of disease, family history, previous medical history, drug use, smoking, and drinking history.

#### 2.8.2 Primary outcomes

The primary outcome is change in visceral fat area (VFA) and TCM syndromes efficacy score scale. Changes in VFA will be compared between the two groups before and after treatment. The indicator will be tested at baseline and week 12. The TCM symptom scoring standard is presented in Supplementary Material S3.

#### 2.8.3 Secondary outcomes

1) Changes in hepatic steatosis factors by ultrasound grading of steatosis.
2) Changes in metabolic parameters: glycated hemoglobin (HbA1c) levels, fasting plasma glucose (FPG), 2-h plasma glucose (2h-PG), fasting insulin, blood lipids including total cholesterol (TC), triglycerides (TG), high-density lipoprotein-cholesterol (c-HDL), low-density lipoprotein (c-LDL), apolipoprotein A (ApoA), and apolipoprotein B (ApoB).
3) Changes in anthropometric parameters: Waist-to-height ratio (WHtR), Body Mass Index (BMI), waist circumference (WC), hip circumference (HC).
4) Changes in hepatic fibrosis factors and hepatic function: The changes in Fibrosis 4 Score (FIB-4, http://gihep.com/calculators/hepatology/fibrosis-4-score/) can be calculated by the formula:

$$\mathrm{Age}\;\left(\mathrm{years}\right)\text{x}\;\mathrm{AST}\;/\mathrm{PLT}\;\left(\times 10^{9}/L\right)\text{x}\sqrt{\mathrm{ALT}}$$

The units of measurement for ALT and AST are aligned with those for age and PLT.

Definitions for the following abbreviations: AST: Aspartate aminotransferase; PLT: platelet; ALT: Alanine aminotransferase.

5) Changes in visceral fat area.

All measurements will be performed at baseline and at week 12. Additional FPG and blood lipid test will be performed at week 6.

#### 2.8.4 Monitoring outcomes

Homeostasis model assessment for insulin resistance (HOMA-IR) and homeostatic model assessment for β-cell function (HOMA-β) will be performed at baseline and at week 12.

#### 2.8.5 Biological specimen analysis outcomes

Untargeted and targeted metabolomics tests will be performed sequentially on fasting blood and urine samples collected at enrollment and at the end of the 3-month treatment period. Biological samples will be centrifuged and stored in a -80°C refrigerator. Assays will be performed using liquid chromatography-mass spectrometry (LC-MS) techniques for microscopic high-throughput measurements. Through basic analysis, multivariate statistical analysis, and difference analysis, the differential metabolites in serum and urine, before and after treatment, will be compared between the two groups. Further targeted metabolomic analyses will be performed to categorize differential metabolites screened by untargeted metabolomics, as well as explore their possible metabolic pathways. The exploratory results will be combined with clinical efficacy evaluation to determine the mechanism of action of JTTF in the treatment of T2DM complicated with NAFLD from the perspective of metabolomics and small-molecule compounds.

#### 2.8.6 Safety outcomes and adverse events

Safety assessments including vital signs, routine blood and urine tests, 12-lead electrocardiogram, and hepatic function (AST/ALT/GGT) and renal function tests will be conducted. General vital signs (body temperature, heart rate, respiration rate, and blood pressure) will be assessed at baseline and at weeks 4, 6, 8, and 12. Routine blood and urine tests will be performed at baseline and weeks 6 and 12.

An adverse event (AE) is defined as any adverse medical event that occurs between the time the participant signs the informed consent and the last follow-up visit, whether or not it is causally related to the study drug. Adverse events, such as subjective patient discomfort and abnormalities in laboratory tests will be carefully analyzed and investigated during each follow-up visit. Overall, JTTF is relatively safe and may be associated with mild diarrhea. If this occurs, consider taking half the dose for 2 days. Absence of remission or exacerbation may be reported to the Principal Investigator, unit management, and the Ethics Committee after evaluation, followed by completion of an AE reporting form. All adverse events will be followed until the condition is stable (discontinuation of observation is considered medically acceptable) or complete resolution has been achieved.

### 2.9 Randomization and data collection

Patients successfully enrolled in the study will be randomly assigned to either the treatment or control group at a 1:1 ratio. The randomization sequence number will be sealed in an opaque envelope and provided only to clinicians so that the participants can be assigned to the treatment regimen accordingly. Since this trial is an open-label study, participants and physicians will be aware of the assignment protocol, whereas data analysts and statisticians will not be aware of the distribution results.

In this study, all participants’ data will be collected, recorded, and administered in the CRF provided by the Ethics Committee of Shenzhen Hospital of Guangzhou University of Chinese Medicine (Futian). Data from the original CRF and biological samples will be stored for an additional 5 years after the trial ends.

### 2.10 Sample size calculation

In a previous study (Xu, 2017), JTTF reduced HbA1c levels in diabetic patients with heat stagnation in liver and stomach after 12 weeks of treatment (treatment group: 6.94±0.88%, control group: 7.64±0.72%, mean difference between the two groups: 0.7%). Considering a type I error of 5% (α = 0.05) and a power of 85%, the experimental and control groups were allocated at a ratio of 1:1. The sample size was calculated using Power Analysis and Sample Size (PASS) software (version 15.0); a total of 76 patients will be required. According to the dropout rate of 20%, we set the final sample size to 96, with 48 patients in each group, considering the number of dropout samples.

### 2.11 Recruitment

Participants in this clinical trial will be recruited mainly from outpatients and inpatients of the Endocrinology Department of the Guangzhou University of Chinese Medicine Shenzhen Hospital (Futian). Patients will also be recruited through posters and publicity on social media platforms. All examinations during the trial will be free of charge to participants. Members of the project team will conduct a detailed protocol explanation and screening of patients who volunteer to participate in the trial to ensure that participants fully understand the trial process and the inclusion criteria.

## 3 Statistical methods

### 3.1 Data analysis

Data will be analyzed using SPSS software (Version 24.0; IBM Corp. Armonk, NY, United States). Continuous variables will be represented as means, standard deviations, medians, and minimum/maximum values. If the data confirms normal distribution, an independent sample *t*-test will be used to assess the differences between the two groups, and a paired sample *t*-test to compare the differences between groups before and after the intervention. Wilcoxon signed-rank test will be used for data that does not conform to normal distribution. Grade data will be analyzed using Wilcoxon’s signed-rank test or the CMHχ2 test. Statistical significance will be set at *p* < 0.05.

### 3.2 Monitoring

To maintain high-quality data and ensure the smooth running of the trial, all protocol implementers and investigators will undergo systematic and rigorous training. Patient screening, data entry, dosing, adverse event reporting, dosing records, and biospecimen extraction will be performed. The data of numbered participants will be recorded in detail on standardized CRF forms and biospecimens will be stored under the same number. Data will be recorded independently by two research assistants, updated in real time, and synchronized on the CRF form after each visit. Quality control and data review will be performed regularly by the study monitoring committee to ensure accuracy and completeness of data entry. The quality and progress of the project will be evaluated. An interim analysis of the primary outcome will be conducted to monitor efficacy and safety when 50% enrollment is reached. This is independent of the sponsor. All patient information and clinical data will be kept confidential on a professional data platform.

## 4 Discussion

NAFLD has a high prevalence worldwide, affecting approximately 25% of the population (Younossi et al., 2021). It is extremely harmful, especially when combined with diabetes, and significantly increases the risk of developing cirrhosis and liver cancer as well as the mortality rate (Golabi et al., 2018; Younossi and Henry, 2019). However, there are still no safe and effective drugs for the treatment of T2DM in combination with NAFLD (Ferguson and Finck, 2021). The current first-line interventions for both NAFLD, and T2DM with NAFLD remains lifestyle modification and weight loss, which also has the disadvantages of low compliance and difficulty in long-term implementation. Therefore, there is a need for a protocol that systematically treats T2DM combined with NAFLD as a comorbid disease, while improving clinical symptoms and combining metabolomics to identify targeted pathways of action to regulate glucose and lipid metabolic abnormalities.

This study is a double-armed, parallel-designed, randomized, controlled clinical trial that aimed to verify the efficacy and safety of JTTF for glucose-lowering and lipid-modulation and to investigate the differences in serum and urine metabolites before and after treatment. The proposed protocol combines clinical evaluation with metabolomics in an attempt to determine its mechanism of action and pathway of action, offering the possibility to systematically elucidate the effectiveness and mechanisms of action of JTTF. Considering the limitations of liver biopsy implementation, this study focused on subjects with fatty liver diagnosed by ultrasound to observe changes in visceral fat and subcutaneous fat area. In addition, patients’ blood lipids, liver function, BMI, HbA1c level, blood glucose, TCM symptom scores, and other indicators will be assessed, and the degree of fibrosis and insulin resistance will be evaluated. Disease onset and progression will be comprehensively evaluated in this clinical study.

This study has some limitations. First, coronavirus outbreaks remain a serious concern worldwide and may affect patient follow-up. In addition, this is a single-center study conducted in China; therefore, the generalizability of the findings remains unclear. Finally, the clinical application of JTTF should be expanded to provide evidence for further mechanistic studies.

## 5 Trial status

This clinical trial has been registered in China Clinical Trial Registry (ChiCTR 2100051174) (https://www.chictr.org.cn/index.aspx **)**. At the time of this manuscript submitted, the state of the trial was at the recruitment stage. Recruitment is expected to be completed by October 2022.
