# Supplementary figures and images for: Efficacy and mechanism of Jiedu Tongluo Tiaogan Formula in treating type 2 diabetes mellitus combined with non-alcoholic fatty liver disease: Study protocol for a parallel-armed, randomized controlled trial
Source: Front Pharmacol. 2022 Aug 12;13:924021. doi: 10.3389/fphar.2022.924021 (PMC9411737; doi:10.3389/fphar.2022.924021)

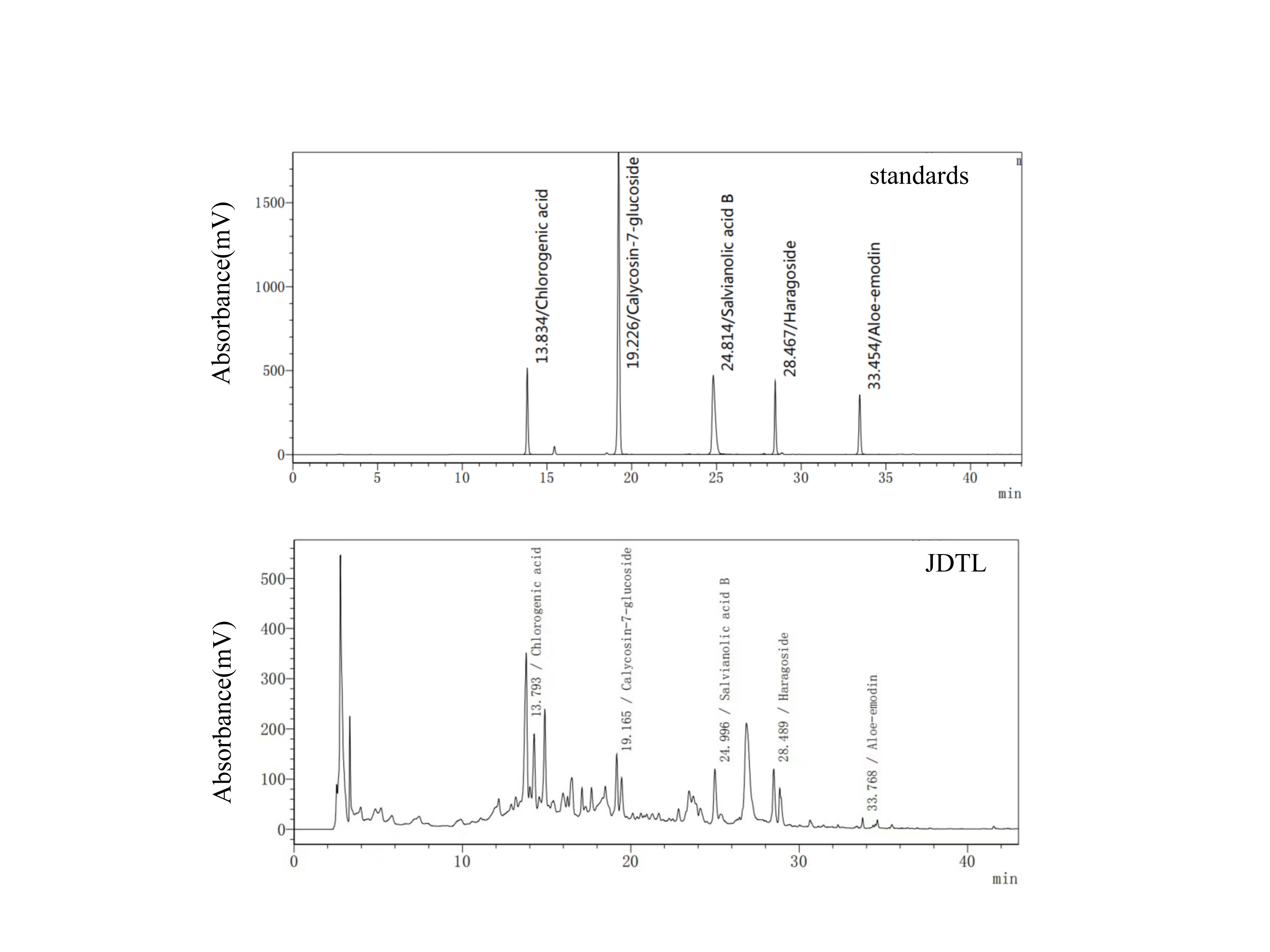

Supplement: Supplementary file 1 [file Image1.JPEG]
