# Supplementary material for: Efficacy and mechanism of Jiedu Tongluo Tiaogan Formula in treating type 2 diabetes mellitus combined with non-alcoholic fatty liver disease: Study protocol for a parallel-armed, randomized controlled trial
Source: Front Pharmacol. 2022 Aug 12;13:924021. doi: 10.3389/fphar.2022.924021 (PMC9411737; doi:10.3389/fphar.2022.924021)
Supplement: Supplementary file 3 [file DataSheet1.docx]

| **Supplementary Material S3** TCM symptoms scoring standard | |
| --- | --- |
| **Main symptoms** | |
| a. Dry mouth | |
| 0 point | Asymptomatic or extremely occasional |
| 2 points | Occasionally feel dry mouth, mild symptoms |
| 4 points | Frequent symptoms of dry mouth |
| 6 points | Throughout the day, with obvious symptoms that are not relieved by drinking water |
| b. Bitter and sticky mouth | |
| 0 point | Asymptomatic or extremely occasional |
| 2 points | Bitter and sticky occasionally |
| 4 points | Frequent symptoms of bitter and sticky mouth |
| 6 points | High frequency and longer duration of bitter and sticky mouth |
| c. Heaviness in head and body | |
| 0 point | Asymptomatic or extremely occasional |
| 2 points | Occasionally feel heaviness |
| 4 points | Frequent symptoms of heaviness, lazy activity |
| 6 points | Heavy and drowsy head and body all day long, sleepiness |
| d. Obesity | |
| 0 point | Standard value |
| 2 points | BMI>25kg/m^2^ |
| 4 points | BMI>30kg/m^2^ |
| 6 points | BMI>35kg/m^2^ |
| **Secondary symptoms** | |
| a. Fullness and distention in the chest or abdomen | |
| 0 point | Asymptomatic or extremely occasional |
| 1 point | Occasionally feel fullness and disetention in the chest or abdomen, half an hour can relieve |
| 2 points | Frequent symptoms, two hours can relieve |
| 3 points | Persistent and difficult to alleviate |
| b. Upset or irritable | |
| 0 point | Asymptomatic or extremely occasional |
| 1 point | Occasionally upset or irritable |
| 2 points | Frequent symptoms |
| 3 points | Persistent and difficult to alleviate, affects sleep and life |
| c. Rapid digestion of food and polyorexia | |
| 0 point | Asymptomatic or extremely occasional |
| 1 point | Occasionally occurrence, increase in food intake by 0.5 times or less |
| 2 points | Frequent symptoms, 0.5 times increase in food consumption |
| 3 points | High frequency, more than 1 time increase in food consumption |
| d. Deep-colored or turbid urine | |
| 0 point | Asymptomatic or extremely occasional |
| 1 point | Occasionally occurrence, no urinary discomfort |
| 2 points | Frequent, with discomfort in urination |
| 3 points | Persistent, with discomfort in urination |
